# How does multiple sclerosis affect sexual satisfaction in patients' spouses?

**DOI:** 10.3389/fpsyg.2023.1110884

**Published:** 2023-04-04

**Authors:** Behnaz Sedighi, Maryam Abedini Parizi, Ali Akbar Haghdoost, Parya Jangipour Afshar, Hamid Reza Shoraka, Simin Jafari

**Affiliations:** ^1^Neurology Research Center, Kerman University of Medical Sciences, Kerman, Iran; ^2^Epidemiology, Modeling in Health Research Center, Institute for Futures Studies in Health, Kerman University of Medical Sciences, Kerman, Iran; ^3^Department of Biostatistics and Epidemiology, Faculty of Public Health, Kerman University of Medical Sciences, Kerman, Iran; ^4^Vector-Borne Diseases Research Center, North Khorasan University of Medical Sciences, Bojnurd, Iran

**Keywords:** multiple sclerosis, sexual satisfaction, spouses, quality of life, Iran

## Abstract

**Background:**

Sexual dysfunction (SD) is a common complaint among multiple sclerosis (MS) patients with a significant impact on the quality of life (QoL) of afflicted couples. The purpose of this study was to determine sexual satisfaction (SS) in the spouses of MS patients and its impact on the QoL.

**Methods:**

A total of 214 spouses of MS patients were enrolled in this cross-sectional study. They completed the Larson Sexual Satisfaction Questionnaire and SF-8 Health Survey.

**Results:**

The mean ± SD age of the spouses was 39.8 ± 9.7 years, and the duration of MS was 5 years or less in most of their partners. The mean ± SD score of QoL was 71.0 ± 20.3 (out of 100), and the mean SS score was 89.2 ± 18.6 (out of 125), showing moderate satisfaction. The highest score was among male spouses younger than 40 years old. The SS scores were also lower among female spouses. In the final model, it was found that SD, psychiatric symptoms, cognitive impairment, and the level of disability of patients were independent explanatory factors for the SS of their spouses.

**Conclusion:**

The findings supported the role of SS in the QoL of spouses of MS patients. Therefore, the attention of physicians to this hidden aspect of the life of MS patients is crucial.

## Introduction

Multiple sclerosis (MS), one of the most disabling central nervous system (CNS) diseases, is an autoimmune inflammatory demyelinating pathology, affecting about 2.3 million people worldwide. Based on the location of plaques, their symptoms vary, such as acute optic neuritis, fatigue, weakness, cognitive and psychiatric disorders, sensational symptoms, sphincter disorders, and sexual dysfunction (SD) (Qaderi and Khoei, 2014; Giesser, 2015; Ayache and Chalah, 2017).

Sexual dysfunction is one of the most common symptoms in MS patients and may arise in all phases of the sexual response cycle (libido, arousal, orgasm, and relaxation) (Foley, 2015). Several studies on SD have shown various forms of SD, including decreased perineal sensation, libido, and vaginal lubrication in women, and difficulty achieving orgasm in both genders (Schmidt et al., 2005; Marck et al., 2016; Petersen et al., 2020).

Sexual dysfunction in MS patients and their spouses is attributed to primary, secondary, and even tertiary complications including hormonal imbalance, demyelinating changes in the brain and spinal cord (specifically the S_2 − 4_ region that causes sexual, bladder, and bowel problems), pain, muscle weakness, fatigue, and spasticity. MS also has psychological, emotional, social, and cultural aspects that interfere with sexual feelings and experiences (Foley et al., 2001, 2013; Qaderi and Khoei, 2014; Vitkova et al., 2014).

In addition, SD is highly correlated with the general perception of the quality of life (QoL) in couples in general and specifically in couples affected by MS. For instance, intestinal and bladder dysfunctions, which are correlated with sexual disorders, reduce the QoL. A study on a large group of MS patients showed that SD affects the mental aspect of QoL (Foley, 2015). This indicates the importance of screening and treatment of sexual disorders for enhancing the QoL in couples afflicted by MS (Vitkova et al., 2014; Foley, 2015). However, limited studies have addressed sexual satisfaction (SS) and its possible effects on QoL in the spouses of MS patients, particularly in Iran with an increasing trend in the prevalence of this disease (Lublin and Miller, 2008; Riley and Tullman, 2010; Eskandarieh et al., 2017). Therefore, this study aimed to identify the scope of SS and its effect on the QoL of spouses of MS patients.

## Methods

### Study design and participants

This cross-sectional study aimed to evaluate the QoL and SS in the spouses of MS patients in the only referral clinic in Kerman, one of the largest cities in Iran, from May 2019 to April 2020. Based on the records of MS patients in the registry of the clinic, the final diagnosis of MS in this registry was confirmed by neurologists working at Shafa Hospital affiliated with the Kerman University of Medical Sciences, based on the revised McDonald criteria (2017) (Thompson et al., 2018). In this phase, those patients and their spouses who had chronic comorbidities (such as diabetes, congestive heart failure, psychiatric or mental disorder, and genitourinary pathologies), those who were not sexually active during the past 6 months, and those who did not agree to participate were excluded. There were 347 married cases, and 214 (0.61) people who met the eligibility were recruited for the study.

### Outcomes measurement

After obtaining written informed consent forms from the patients and their spouses, data were collected using four instruments: (1) A demographic and clinical information form to assess the spouse's sex, age, education, job, income, duration of the marriage, and the patient's clinical conditions including the duration of disease, sphincter disorder, psychiatric symptoms, cognitive impairment, and SD, (2) the Expanded Disability Status Scale (EDSS), (3) Larson Sexual Satisfaction Questionnaire, and (4) SF-8 Health Survey.

The Sexual Satisfaction Questionnaire was designed by Larson et al. (1998) to measure the level of SS. The questionnaire consists of 25 items scored on a 5-point Likert scale (1 = not at all, 2 = seldom, 3 = sometimes, 4 = often, and 5 = always). The total score ranges from 25 to 125. Scores below 50 represent sexual dissatisfaction, 51–75 low satisfaction, 76–100 moderate satisfaction, and more than 101 high satisfaction (Larson et al., 1998). The reliability of the Persian version of the questionnaire was assessed and confirmed by Bahrami et al. (2016), with Cronbach's alpha of 0.70 and internal consistency of *r* = 0.93 (Bahrami et al., 2016). Furthermore, Cronbach's reliability coefficients of the SS Questionnaire in our study were 0.91.

The SF-8 Health Survey was developed by Ware in 2001 (Ware, 2001) consisting of eight domains, including general health, physical functioning, physical role, bodily pain, vitality, social functioning, mental health, and emotional roles. Four domains (general health, physical functioning, physical role, and bodily pain) measure the physical aspect of the QoL, and the other four domains (vitality, social functioning, mental health, and emotional roles) depict the mental aspect of the QoL. The total score ranges from 0 to 100. The reliability of the SF-8 Health Survey was 0.89 among the Iranian population. (Ghafari et al., 2009). In addition, Cronbach's reliability coefficients for the SF-8 Health Survey in our data were 0.89.

The Expanded Disability Status Scale (EDSS) of MS patients was designed by John Kurtzke. It assesses the functioning of systems such as pyramidal, cerebellar, brainstem, sensory, bowel, bladder, visual, and cerebellar regions (Kurtzke, 1983). The total score on this scale varies from 0 (normal neurological state) to 10 (MS-induced death).

All questionnaires were filled by the spouses in a calm and private environment under the supervision of a neurologist, and the EDSS was assessed by a neurologist. The records were kept and analyzed anonymously.

### Statistical analysis

The scores of the participants in every questionnaire were computed based on their guidelines. Having described scores, all variables were modeled using a linear regression model. Multivariable linear regression analysis was conducted on the variables with a *p*-value of < 0.2 in univariable regression analysis (Maldonado and Greenland, 1993; Dohooet al., 2012) to evaluate the relationship between the demographic variables in the spouses of MS patients and the clinical details of the patients with the mean±SD score of SS in the spouses. The goodness of fit of the model with the score of SS (R^2^=0.67) in the spouses was acceptable. This statistic indicates that 67% of the variation in SS is explained by demographic variables in the spouses of MS patients and the clinical information of MS patients. The correlation between QOL and SS scores was evaluated by Pearson's correlation coefficient. The data were analyzed using SPSS software (version 22), and p-values of less than 0.05 were considered statistically significant.

### Ethics statement

This study was approved by the Ethics Committee of Afzalipour Hospital, Kerman University of Medical Sciences with approval ID: IR.KMU.AH.REC.1398.072. Written informed consent was obtained from all the participants.

## Results

### Clinical and sociodemographic characteristics

In this study, 214 spouses with a mean ± SD age of 39.8 ± 9.7 years (range: 20–67 years) participated. The demographic data of the spouses (sex, age, education, job, income, and duration of marriage) and the clinical details of the patients (duration of disease, EDSS, sphincter disorder, psychiatric symptoms, cognitive impairment, and SD) are shown in Table 1. More than 75% of the participants were men, 57.1% were younger than 40 years, and 81% had 12 or more years of formal education.

**Table 1:** Demographic variables of the spouses of multiple sclerosis (MS) patients and the clinical details of MS patients.

**Variables**			**Frequency**	**Percent**
**Participants (spouses of MS patients)**				
Sex	Male		162	75.7
	Female		52	24.3
Age	Less than 40		120	57.1
	40 or more		90	42.9
Education (years of formal education)	<12		40	19
	12–16		146	69.5
	>16		24	11.5
Job	Unemployed		55	25.8
	Self-employed		80	37.6
	Employed		78	36.6
Income	very low		37	17.7
	Low		92	44
	average		57	27.3
	higher than average		23	11
The duration of marriage (year)	<= 10		84	39.4
	11–20		75	35.2
	21–30		43	20.2
	> 30		11	5.2
**MS patients**				
The duration of MS (year)	<= 5		113	54.5
	6–10		60	29
	11–15		28	13.5
	16–20		6	3
EDSS	Mild (0–2.5)		152	71.7
	Moderate (3–5)		42	19.8
	Sever (5.5–10)		18	8.5
Main symptoms	Sphincter disorder	No	203	94.9
		Yes	11	5.1
	Psychiatric symptoms	No	120	56.1
		Yes	94	43.9
	Cognitive impairment	No	184	86.0
		Yes	30	14.0
	Sexual dysfunction	No	181	84.6
		Yes	33	15.4

### QoL and SS information

The mean ± SD QoL score was 71.0 ± 20.3 out of 100. The highest and the lowest mean QoL scores were related to the physical functioning subscale (81.3) from physical QoL and the vitality subscale (60.3) from mental QoL, respectively. The mean ± SD SS score was 89.2 ± 18.6 out of 125, showing moderate satisfaction, and the mean EDSS score was 1.7 out of 10, which was categorized as mild (Table 2).

**Table 2:** Mean and standard deviation of QoL and its dimensions, as well as sexual satisfaction in spouses and EDSS in MS patients.

**Variables**		**Mean**	**Standard deviation**
QoL		71.0	20.3
Physical QoL		73.1	22.2
Mental QoL		68.9	21.6
QoL subscales	general health	62.2	24.3
	physical functioning	81.3	25.3
	physical role	78.4	27.3
	bodily pain	70.7	29.8
	vitality	60.3	24.9
	social functioning	77.0	28.2
	mental health	60.4	29.9
	emotional roles	78.2	23.5
Sexual Satisfaction	overall	89.2	18.6
	Male	90.7	17.6
	Female	84.6	20.9
EDSS		1.7	1.7

EDSS, Expanded Disability Status Scale; QoL, quality of life.

### Factors associated with SS

As shown in Table 3, the SS mean score (93.1 ± 16.1) in spouses younger than 40 was higher than those aged 40 or more (84.0 ± 20.7). The SS mean score was lower in women (84.7 ± 20.9) than in men (90.7 ± 17.6), but there was no statistically significant difference in SS (*P* = 0.2 and 0.06).

**Table 3:** Results of linear regression models and SS of spouses were linked (as dependent variables) to demographic variables, and the clinical presentations of the MS cases.

**Variables**			**Sexual satisfaction**		
			**Mean ±SD**	**Multiple regression**	
				**coefficient**	**P-value**
**Participants (spouses of MS patients)**					
Sex	Male		90.7(±17.6)	Ref. group
	Female		84.7(±20.9)	−1.7	0.06
Age	Less than 40		93.1(±16.1)	Ref. group
	40 or more		84.1(±20.7)	−3.6	0.27
Education (years of formal education)	<12		82.6(±23.8)	Ref. group
	12–16		89.9(±17.2)	2.6	0.41
	>16		97.3(±13.3)	3.7	0.42
Job	Unemployed		84.4(±19.5)	Ref. group
	Self-employed		91.9(±17.9)	5.5	0.08
	Employed		89.3(±18.7)	1.4	0.68
Income	Very low		84.1(±21.8)	Ref. group
	Low		85.7(±19.2)	−0.3	0.92
	average		93.1(±14.7)	5.8	0.12
	higher than average		100.9(±12.8)	9.0	0.06
The duration of marriage (year)	<=10		94.6(±13.1)	Ref. group
	11–20		89.6(±19.8)	1.6	0.59
	21–30		82.2(±21.8)	−1.2	0.78
	>30		(16.9±)69.4	−8.5	0.18
**MS patients**					
The duration of MS (year)	< =5		92.6(±15.6)	Ref. group
	6–10		85.8(±22.6)	−5.1	0.04^*^
	11–15		87.1(±19.3)	0.1	0.97
	16–20		81.8(±13.3)	0.4	0.95
EDSS	Mild (0–2.5)		93.6(±16.2)	Ref. group
	Moderate (3–5)		79.0(±20.2)	−6.9	0.02^*^
	Sever (5.5–10)		73.3(±20.2)	−11.6	0.04^*^
Symptoms	Sexual dysfunction	No	92.1(±17.1)	Ref. group
		Yes	73.5(±19.0)	−12.8	< 0.0001^*^
	Sphincter disorder	No	90.1(±18.2)	Ref. group
		Yes	73.2(±19.4)	−0.8	0.88
	Psychiatric symptoms	No	92.4(±18.3)	Ref. group
		Yes	85.3(±18.3)	−6.0	0.008^*^
	Cognitive impairment	No	91.5(±17.4)	Ref. group
		Yes	75.4(±20.3)	−7.7	0.01^*^

* *P* < 0.05 is significant.

According to the regression results, a significant inverse relationship between EDSS and SS was found. It means that MS patients with moderate and severe EDSS had lower SS than those with mild EDSS (B = −6.9, *P* < 0.05 and B=-11.6, *P* < 0.05, respectively).

Patients with psychiatric symptoms, cognitive impairment, sphincter disorder, and sexual dysfunction had a low mean SS score. Furthermore, according to the results of the regression models, sexual dysfunction, psychiatric symptoms, and cognitive impairment significantly reduced the SS score (*P* < 0.05).

### Correlation between QoL and SS

The Pearson correlation test was used to evaluate the correlation between the QoL and SS scores. There was a significant positive correlation between SS and QoL in the spouses of MS patients (*P* < 0.05) (Table 4).

**Table 4:** Correlation between sexual satisfaction and quality of life (QoL).

**Variable**	**Sexual satisfaction**	
	**Correlation coefficient**	**P-value**
QoL	0.530	<0.0001^*^
Physical QoL	0.499	<0.0001^*^
Mental QoL	0.469	<0.0001^*^

* Correlation is significant at *P* < 0.05.

## Discussion

This study investigated SS, QoL, and their relationship in the spouses of MS patients in Kerman Province, Iran. The results showed that SS and QoL were lower than the general population. In addition, an assessment of the factors associated with SS showed that EDSS, psychiatric symptoms, cognitive impairment, and SD were significant in MS patients. Moreover, there was a significant positive correlation between QoL and SS in the spouses of MS patients.

In the current study, the participants' SS score was 89 out of 125, which was lower than the scores in the overall Iranian population reported by Rahmani et al., Amiri et al., and Bahrami et al. According to these reports, sexual satisfaction was high in men and moderate to high in women, whereas this study found SS to be moderate in both genders (Rahmani et al., 2010; Bahrami et al., 2016; Amiri et al., 2020).

Although the female partners had lower SS scores compared to the male partners in this study, it is unclear whether this was merely due to the difference in the presentation of MS in male and female patients. Studies in Iran found that SS was lower in females than in males in the general population (Bahrami et al., 2016). Therefore, the observed difference between male and female spouses cannot be attributed to the impact of MS only.

The results of this study in terms of sexual satisfaction were consistent with many chronic diseases including inflammatory bowel disease, cancer, diabetes mellitus, and hypertension (Eluri et al., 2018; Szydlarska et al., 2019; Umrigar and Mhaske, 2022). This could be due to lower QoL, depression, anxiety, the psychological impact of having a chronic illness, disease activity, and sexual disorders caused by the disease. In addition, numerous studies have shown that disability and chronic diseases can be associated with low self-esteem. Low self-esteem develops a sense of low self-confidence in oneself to have an effective and desirable sexual activity, leading to low sexual satisfaction (Delaney and Donovan, 2017; Alirezaei and Ozgoli, 2018).

In this study consistent with other studies, a significant relationship between SS in spouses and SD in MS patients was detected (Schmidt et al., 2005; Foley, 2015; Marck et al., 2016; Petersen et al., 2020). SD in MS patients is a complex and multidimensional disorder, in which decreased perineal sensation, libido, and vaginal lubrication could decrease the orgasmic response, which has a significant negative effect on sexual satisfaction in both the patients and their spouses (Darija et al., 2015; Foley, 2015; Marck et al., 2016; Petersen et al., 2020).

In this study, a significant negative association was found between psychiatric symptoms in MS patients and spouses' SS, as evident in other studies (Mohammadi et al., 2013; Giesser, 2015; Petersen et al., 2020). A longitudinal study in Belgrade showed that depression, anxiety, and fatigue all have an impact on SD in MS patients (Darija et al., 2015). Due to the progressive nature of MS, physical and psycho-mental disorder increase in patients over time, leading to a decrease in SS. Psychological and social consequences of MS such as depressive mood, negative attitudes toward body image, and lower self-assurance can adversely affect sexual functioning and inhibit orgasm (Darija et al., 2015; Giesser, 2015; Petersen et al., 2020). Therefore, screening and treatment of depression and psychiatric problems and also counseling programs are recommended for couples with MS, especially in Iran.

The results also indicated a negative association between EDSS and SS, which was similar to other previous studies (Qaderi and Khoei, 2014; Vitkova et al., 2014). It shows that the aggravation of physical impairment can exacerbate sexual problems among couples with MS. Furthermore, muscle tightness, body spasms, and physical inability can affect the sexual activity and decrease sexual satisfaction in these couples (Qaderi and Khoei, 2014; Giesser, 2015).

The patients' problems in a sexual relationship, including dyspareunia, bladder and bowel problems, muscle weakness, fatigue, and spasticity, as well as psychological, emotional, social, and cultural factors (body image concern and sexual performance anxiety in the patient and reduced self-esteem because of sexual disability and inhibition) (Qaderi and Khoei, 2014; Foley, 2015; Giesser, 2015; Marck et al., 2016; Petersen et al., 2020) may result in reduced sexual relationships and SS. Overall, SS in the spouses of MS patients seems to be lower than in the normal population.

This study also found a significant positive correlation between QoL and SS in spouses of MS patients, as indicated in other studies (Nortvedt et al., 2007; Tompkins et al., 2013). Norvedt et al. found that SS has a vigorous relationship with QoL in MS patients (Nortvedt et al., 2007). Tompkins et al. found that a relationship enrichment program for both MS patients and their spouses could change their attitudes and psychiatric disorders caused by MS and, as a result, improve their QoL and SS (Tompkins et al., 2013). Chronicity of the disease and the mentioned parameters lead to the change in the spouse's role as a caregiver over time. In addition, in the Iranian culture, a sexual relationship is a monogamous relationship that starts just after marriage, so if one of the couples has a sexual disorder, it will cause sexual dissatisfaction on both sides and even can influence the continuation of marital life.

Furthermore, Iranian people and even physicians often feel ashamed of talking about sexual problems. Thus, people with such problems often have difficulty consulting with health professionals and seeking treatment or support (Rezaei et al., 2021).

In addition, as the sexual problems of MS patients and their spouses are usually missed in the treatment procedure and, thus, they do not receive suitable treatment for their sexual issues, and active screening for the diagnosis of sexual dissatisfaction in MS couples is recommended.

Similarly, fatigue, anxiety, and depression of MS patients affect their spouses' SS. Hence, these patients should resolve these symptoms to improve their sexual relationships. Furthermore, non-pharmacological strategies, including appropriate sexual positions and sexual intercourse at times of feeling more energized, should be considered in the process of consulting MS couples (Foley, 2015; Zamani et al., 2017).

These results show that the MS of partners might change the SS score not substantially. However, for more reliable results, a more comprehensive study is recommended to recruit normal and MS couples simultaneously.

## Conclusion

Sexual satisfaction and its impact on the quality of life of MS patients and their partners are crucial issues in Iran. Cultural barriers and shame around speaking about sexual experiences limit the effective communication between patients and spouses with their physicians. Therefore, special attention should be paid to this issue.

## Data availability statement

The raw data supporting the conclusions of this article will be made available by the authors, without undue reservation.

## Ethics statement

The studies involving human participants were reviewed and approved by the Ethics Committee of Afzalipour Hospital, Kerman University of Medical Sciences, approval ID; IR.KMU.AH.REC.1398.072. Written informed consent has been obtained from all the participants. The patients/participants provided their written informed consent to participate in this study.

## Author contributions

BS wrote the proposal and the manuscript. MAP wrote the proposal, analyzed data, and wrote the manuscript. AAH, PJA, and HRSH analyzed data and wrote the manuscript. SJ wrote the proposal, collected data, analyzed data, and wrote the manuscript, and corresponding author. All authors read and approved the manuscript.
